# Bis(hexa­methyl­enetetra­mine)bis­(tri­chloro­acetato)copper(II)

**DOI:** 10.1107/S1600536809043736

**Published:** 2009-10-31

**Authors:** Li-Min Li, Fang-Fang Jian, Yu-Feng Li

**Affiliations:** a Microscale Science Institute, Department of Chemistry and Chemical Engineering, Weifang University, Weifang 261061, People’s Republic of China; b Microscale Science Institute, Weifang University, Weifang 261061, People’s Republic of China

## Abstract

In the title compound, [Cu(C_2_Cl_3_O_2_)_2_(C_6_H_12_N_4_)_2_], the Cu^II^ ion (site symmetry 2) is coordinated by two trichloro­acetate anions and two hexa­methyl­enetetra­mine mol­ecules, resulting in a distorted CuN_2_O_2_ geometry that is inter­mediate between tetra­hedral and square planar. The Cl atoms are disordered over two sets of sites, with relative occupancies of 0.749 (7) and 0.251 (7). In the crystal, the packing is consolidated by inter­molecular C—H⋯O inter­actions.

## Related literature

For background to coordination networks, see: Chen *et al.* (2001 ▶). For a related structure, see: Moncol *et al.* (2007 ▶).
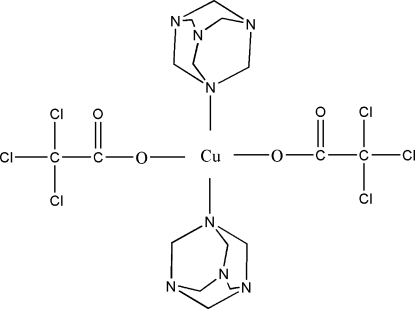


## Experimental

### 

#### Crystal data

[Cu(C_2_Cl_3_O_2_)_2_(C_6_H_12_N_4_)_2_]
*M* *_r_* = 668.67
Monoclinic, 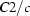

*a* = 23.291 (5) Å
*b* = 6.4759 (13) Å
*c* = 20.702 (4) Å
β = 121.36 (3)°
*V* = 2666.3 (9) Å^3^
*Z* = 4
Mo *K*α radiation
μ = 1.46 mm^−1^
*T* = 293 K
0.30 × 0.20 × 0.15 mm

#### Data collection

Bruker SMART CCD diffractometer
Absorption correction: none
12444 measured reflections
3048 independent reflections
2740 reflections with *I* > 2σ(*I*)
*R*_int_ = 0.023

#### Refinement

*R*[*F* ^2^ > 2σ(*F* ^2^)] = 0.057
*wR*(*F* ^2^) = 0.184
*S* = 1.09
3048 reflections
187 parameters
78 restraints
H-atom parameters constrained
Δρ_max_ = 1.52 e Å^−3^
Δρ_min_ = −0.98 e Å^−3^

### 

Data collection: *SMART* (Bruker, 1997 ▶); cell refinement: *SAINT* (Bruker, 1997 ▶); data reduction: *SAINT*; program(s) used to solve structure: *SHELXS97* (Sheldrick, 2008 ▶); program(s) used to refine structure: *SHELXL97* (Sheldrick, 2008 ▶); molecular graphics: *SHELXTL* (Sheldrick, 2008 ▶); software used to prepare material for publication: *SHELXTL*.

## Supplementary Material

Crystal structure: contains datablocks global, I. DOI: 10.1107/S1600536809043736/hb5145sup1.cif

Structure factors: contains datablocks I. DOI: 10.1107/S1600536809043736/hb5145Isup2.hkl

Additional supplementary materials:  crystallographic information; 3D view; checkCIF report

## Figures and Tables

**Table:** 

Cu1—O1	1.941 (3)
Cu1—N1	2.045 (2)

**Table:** 

O1—Cu1—O1^i^	159.95 (17)
O1—Cu1—N1	89.63 (10)
O1^i^—Cu1—N1	96.49 (11)
N1^i^—Cu1—N1	144.38 (14)

Symmetry code: (i) 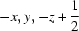.

**Table 2:** Hydrogen-bond geometry (Å, °)

*D*—H⋯*A*	*D*—H	H⋯*A*	*D*⋯*A*	*D*—H⋯*A*
C1—H1*A*⋯O2^ii^	0.97	2.52	3.416 (5)	153

Symmetry code: (ii) 
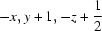
.

**Fig. 1.**
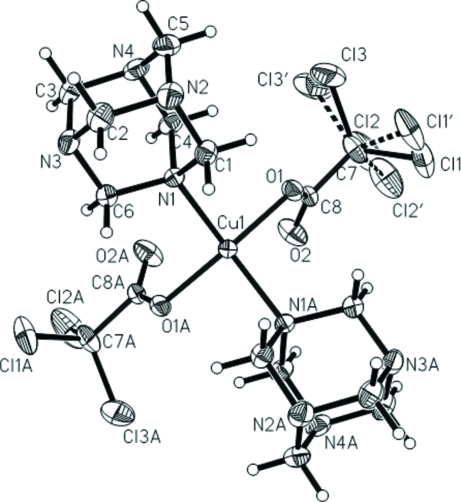
The structure of (I) showing 30% probability displacement ellipsoids. Atoms with suffix A are generated by the symmetry operation (–x, y, 1/2–z).

## supplementary crystallographic information

### Comment

Metal-organic framework coordination polymers have attracted tremendous attention because of their molecular topologies and their potentially useful ionexchange,adsorption,catalytic and magnetic properties. Much of this work has been concerned (e.g. Chen *et al.*, 2001). In order to search for new complexes of this type, we synthesized the title compound, (I), and report its crystal structure here.

The title structure contains one copper(II), two N atoms of the hexamethylenetetramine ligands and two O atoms of trichloroacetate anions. The coordination sphere of the copper(II) ion is best described as a seriously distorted tetrahedral. The Cu—O and Cu—N bond lengths are in agreement with those reported recently (Moncol *et al.*, 2007). The Cl atoms are disordered over two sites, with relatives occupancies 0.749 (7) and 0.251 (7).The crystal packing is stabilized by intra- and intermolecular C—H···O hydrogen interaction (Table 1).

### Experimental

The title compound was obtained by adding hexamethylenetetramine (2 mmol) dropwise to a solution of trichloroacetatocopper(II) (1 mmol) in ethanol (30 ml) under stirred for 1 h at room temperature. A green solution was formed and after a few days block crystals precipitated.

### Refinement

H atoms were fixed geometrically and allowed to ride on their attached atoms, with C—H and N—H distances of 0.93–0.96 and 0.86 Å, and with *U*_iso_ = 1.2*U*_eq_.

### Crystal data

**Table:**

[Cu(C_2_Cl_3_O_2_)_2_(C_6_H_12_N_4_)_2_]	*F*(000) = 1356
*M**_r_* = 668.67	*D*_x_ = 1.666 Mg m^−^^3^
Monoclinic, *C*2/*c*	Mo *K*α radiation, λ = 0.71073 Å
*a* = 23.291 (5) Å	Cell parameters from 2740 reflections
*b* = 6.4759 (13) Å	θ = 3.3–27.5°
*c* = 20.702 (4) Å	µ = 1.46 mm^−^^1^
β = 121.36 (3)°	*T* = 293 K
*V* = 2666.3 (9) Å^3^	Block, green
*Z* = 4	0.30 × 0.20 × 0.15 mm

### Data collection

**Table:**

Bruker SMART CCD diffractometer	2740 reflections with *I* > 2σ(*I*)
Radiation source: fine-focus sealed tube	*R*_int_ = 0.023
graphite	θ_max_ = 27.5°, θ_min_ = 3.3°
Detector resolution: 3 pixels mm^-1^	*h* = −30→30
ω scans	*k* = −7→8
12444 measured reflections	*l* = −26→26
3048 independent reflections	

### Refinement

**Table:**

Refinement on *F*^2^	Primary atom site location: structure-invariant direct methods
Least-squares matrix: full	Secondary atom site location: difference Fourier map
*R*[*F*^2^ > 2σ(*F*^2^)] = 0.057	Hydrogen site location: inferred from neighbouring sites
*wR*(*F*^2^) = 0.184	H-atom parameters constrained
*S* = 1.09	*w* = 1/[σ^2^(*F*_o_^2^) + (0.1275*P*)^2^ + 3.9764*P*] where *P* = (*F*_o_^2^ + 2*F*_c_^2^)/3
3048 reflections	(Δ/σ)_max_ = 0.042
187 parameters	Δρ_max_ = 1.52 e Å^−^^3^
78 restraints	Δρ_min_ = −0.98 e Å^−^^3^

### Special details

**Table:**

Geometry. All e.s.d.'s (except the e.s.d. in the dihedral angle between two l.s. planes) are estimated using the full covariance matrix. The cell e.s.d.'s are taken into account individually in the estimation of e.s.d.'s in distances, angles and torsion angles; correlations between e.s.d.'s in cell parameters are only used when they are defined by crystal symmetry. An approximate (isotropic) treatment of cell e.s.d.'s is used for estimating e.s.d.'s involving l.s. planes.
Refinement. Refinement of *F*^2^ against ALL reflections. The weighted *R*-factor *wR* and goodness of fit *S* are based on *F*^2^, conventional *R*-factors *R* are based on *F*, with *F* set to zero for negative *F*^2^. The threshold expression of *F*^2^ > σ(*F*^2^) is used only for calculating *R*-factors(gt) *etc*. and is not relevant to the choice of reflections for refinement. *R*-factors based on *F*^2^ are statistically about twice as large as those based on *F*, and *R*- factors based on ALL data will be even larger.

### Fractional atomic coordinates and isotropic or equivalent isotropic displacement parameters (Å^2^)

**Table:**

	*x*	*y*	*z*	*U*_iso_*/*U*_eq_	Occ. (<1)
Cu1	0.0000	0.79415 (8)	0.2500	0.0315 (2)	
O1	0.06660 (12)	0.7420 (4)	0.35558 (14)	0.0428 (6)	
O2	0.01619 (16)	0.4397 (5)	0.33908 (17)	0.0669 (9)	
N1	0.07476 (12)	0.8907 (4)	0.23307 (14)	0.0303 (5)	
N2	0.15738 (18)	1.1697 (5)	0.2701 (2)	0.0483 (7)	
N3	0.10588 (16)	1.0194 (5)	0.14402 (17)	0.0445 (7)	
N4	0.18698 (16)	0.8189 (5)	0.2547 (2)	0.0477 (8)	
C1	0.10233 (18)	1.0882 (5)	0.27694 (19)	0.0396 (7)	
H1A	0.0667	1.1900	0.2584	0.047*	
H1B	0.1183	1.0629	0.3299	0.047*	
C2	0.1318 (2)	1.2073 (6)	0.1894 (3)	0.0499 (9)	
H2A	0.0962	1.3096	0.1704	0.060*	
H2B	0.1678	1.2629	0.1840	0.060*	
C3	0.1605 (2)	0.8664 (7)	0.1743 (2)	0.0519 (9)	
H3A	0.1967	0.9186	0.1687	0.062*	
H3B	0.1439	0.7402	0.1449	0.062*	
C4	0.13189 (18)	0.7387 (5)	0.2620 (2)	0.0406 (7)	
H4A	0.1485	0.7085	0.3147	0.049*	
H4B	0.1156	0.6108	0.2337	0.049*	
C5	0.21037 (19)	1.0132 (7)	0.2968 (2)	0.0547 (10)	
H5A	0.2277	0.9861	0.3500	0.066*	
H5B	0.2470	1.0670	0.2924	0.066*	
C6	0.05198 (17)	0.9380 (6)	0.15232 (18)	0.0410 (7)	
H6A	0.0346	0.8129	0.1225	0.049*	
H6B	0.0157	1.0379	0.1328	0.049*	
C7	0.1155 (2)	0.5072 (6)	0.45812 (19)	0.0579 (10)	
C8	0.05989 (16)	0.5630 (6)	0.37587 (17)	0.0378 (7)	
Cl1	0.1084 (2)	0.6786 (5)	0.51998 (15)	0.0872 (9)	0.749 (7)
Cl2	0.1139 (3)	0.2478 (4)	0.48019 (19)	0.1149 (15)	0.749 (7)
Cl3	0.19694 (13)	0.5452 (9)	0.47005 (18)	0.1191 (16)	0.749 (7)
Cl1'	0.1398 (7)	0.7198 (12)	0.5194 (5)	0.102 (2)	0.251 (7)
Cl2'	0.0839 (6)	0.2990 (15)	0.4873 (5)	0.106 (2)	0.251 (7)
Cl3'	0.1851 (4)	0.430 (2)	0.4518 (6)	0.133 (3)	0.251 (7)

### Atomic displacement parameters (Å^2^)

**Table:**

	*U*^11^	*U*^22^	*U*^33^	*U*^12^	*U*^13^	*U*^23^
Cu1	0.0256 (3)	0.0403 (4)	0.0277 (3)	0.000	0.0133 (2)	0.000
O1	0.0349 (12)	0.0555 (14)	0.0316 (12)	−0.0009 (10)	0.0129 (10)	0.0104 (10)
O2	0.0620 (18)	0.0575 (17)	0.0507 (16)	−0.0134 (14)	0.0079 (14)	0.0011 (14)
N1	0.0288 (11)	0.0309 (12)	0.0326 (12)	0.0013 (9)	0.0170 (10)	0.0000 (10)
N2	0.0535 (19)	0.0428 (15)	0.0538 (18)	−0.0155 (14)	0.0316 (16)	−0.0081 (14)
N3	0.0491 (17)	0.0516 (17)	0.0424 (15)	−0.0001 (13)	0.0304 (13)	0.0039 (13)
N4	0.0360 (15)	0.0541 (18)	0.061 (2)	0.0086 (12)	0.0305 (15)	0.0094 (15)
C1	0.0476 (18)	0.0345 (15)	0.0432 (17)	−0.0034 (13)	0.0282 (15)	−0.0068 (14)
C2	0.059 (2)	0.0409 (19)	0.058 (2)	−0.0043 (15)	0.037 (2)	0.0067 (16)
C3	0.059 (2)	0.056 (2)	0.061 (2)	0.0055 (19)	0.046 (2)	0.0001 (19)
C4	0.0395 (17)	0.0361 (15)	0.054 (2)	0.0087 (13)	0.0295 (16)	0.0081 (15)
C5	0.0361 (18)	0.071 (3)	0.053 (2)	−0.0117 (17)	0.0208 (16)	0.0022 (19)
C6	0.0376 (16)	0.0516 (19)	0.0336 (15)	−0.0027 (14)	0.0185 (13)	−0.0008 (14)
C7	0.064 (2)	0.058 (2)	0.0289 (16)	0.0017 (19)	0.0086 (16)	0.0075 (16)
C8	0.0346 (15)	0.0496 (18)	0.0253 (13)	0.0018 (13)	0.0129 (12)	0.0010 (13)
Cl1	0.115 (2)	0.0970 (16)	0.0377 (8)	0.0104 (14)	0.0319 (13)	−0.0064 (9)
Cl2	0.147 (3)	0.0608 (12)	0.0681 (13)	0.0104 (14)	0.0082 (17)	0.0246 (11)
Cl3	0.0462 (11)	0.208 (5)	0.0721 (16)	0.0175 (17)	0.0089 (11)	0.030 (2)
Cl1'	0.131 (5)	0.090 (3)	0.036 (2)	0.008 (3)	0.010 (3)	−0.011 (2)
Cl2'	0.148 (5)	0.064 (3)	0.063 (3)	−0.009 (3)	0.024 (3)	0.026 (3)
Cl3'	0.053 (3)	0.196 (6)	0.092 (4)	0.044 (4)	−0.001 (3)	0.005 (4)

### Geometric parameters (Å, °)

**Table:**

Cu1—O1	1.941 (3)	C1—H1B	0.9700
Cu1—O1^i^	1.941 (3)	C2—H2A	0.9700
Cu1—N1^i^	2.045 (2)	C2—H2B	0.9700
Cu1—N1	2.045 (2)	C3—H3A	0.9700
O1—C8	1.270 (4)	C3—H3B	0.9700
O2—C8	1.203 (5)	C4—H4A	0.9700
N1—C6	1.499 (4)	C4—H4B	0.9700
N1—C4	1.506 (4)	C5—H5A	0.9700
N1—C1	1.505 (4)	C5—H5B	0.9700
N2—C1	1.460 (5)	C6—H6A	0.9700
N2—C5	1.465 (6)	C6—H6B	0.9700
N2—C2	1.473 (6)	C7—C8	1.553 (5)
N3—C6	1.452 (4)	C7—Cl2	1.747 (5)
N3—C2	1.462 (5)	C7—Cl1'	1.754 (7)
N3—C3	1.470 (5)	C7—Cl3'	1.764 (7)
N4—C4	1.463 (5)	C7—Cl1	1.766 (5)
N4—C5	1.465 (6)	C7—Cl2'	1.786 (7)
N4—C3	1.477 (5)	C7—Cl3	1.797 (5)
C1—H1A	0.9700		
			
O1—Cu1—O1^i^	159.95 (17)	N4—C4—H4A	109.3
O1—Cu1—N1^i^	96.49 (11)	N1—C4—H4A	109.3
O1^i^—Cu1—N1^i^	89.63 (10)	N4—C4—H4B	109.3
O1—Cu1—N1	89.63 (10)	N1—C4—H4B	109.3
O1^i^—Cu1—N1	96.49 (11)	H4A—C4—H4B	108.0
N1^i^—Cu1—N1	144.38 (14)	N2—C5—N4	112.9 (3)
C8—O1—Cu1	111.6 (2)	N2—C5—H5A	109.0
C6—N1—C4	107.7 (2)	N4—C5—H5A	109.0
C6—N1—C1	107.0 (3)	N2—C5—H5B	109.0
C4—N1—C1	107.7 (3)	N4—C5—H5B	109.0
C6—N1—Cu1	114.51 (19)	H5A—C5—H5B	107.8
C4—N1—Cu1	112.75 (19)	N3—C6—N1	112.3 (3)
C1—N1—Cu1	106.88 (18)	N3—C6—H6A	109.1
C1—N2—C5	108.8 (3)	N1—C6—H6A	109.1
C1—N2—C2	108.3 (3)	N3—C6—H6B	109.1
C5—N2—C2	107.9 (3)	N1—C6—H6B	109.1
C6—N3—C2	108.7 (3)	H6A—C6—H6B	107.9
C6—N3—C3	108.3 (3)	C8—C7—Cl2	113.0 (3)
C2—N3—C3	108.1 (3)	C8—C7—Cl1'	112.4 (4)
C4—N4—C5	108.6 (3)	Cl2—C7—Cl1'	127.5 (4)
C4—N4—C3	108.4 (3)	C8—C7—Cl3'	105.0 (4)
C5—N4—C3	107.5 (3)	Cl2—C7—Cl3'	83.9 (5)
N2—C1—N1	111.6 (3)	Cl1'—C7—Cl3'	108.3 (5)
N2—C1—H1A	109.3	C8—C7—Cl1	108.0 (3)
N1—C1—H1A	109.3	Cl2—C7—Cl1	113.0 (3)
N2—C1—H1B	109.3	Cl1'—C7—Cl1	25.7 (4)
N1—C1—H1B	109.3	Cl3'—C7—Cl1	131.9 (4)
H1A—C1—H1B	108.0	C8—C7—Cl2'	106.9 (4)
N3—C2—N2	112.3 (3)	Cl2—C7—Cl2'	27.9 (4)
N3—C2—H2A	109.2	Cl1'—C7—Cl2'	112.5 (5)
N2—C2—H2A	109.2	Cl3'—C7—Cl2'	111.6 (5)
N3—C2—H2B	109.2	Cl1—C7—Cl2'	91.0 (4)
N2—C2—H2B	109.2	C8—C7—Cl3	109.7 (3)
H2A—C2—H2B	107.9	Cl2—C7—Cl3	105.1 (3)
N3—C3—N4	112.4 (3)	Cl1'—C7—Cl3	82.7 (5)
N3—C3—H3A	109.1	Cl3'—C7—Cl3	26.5 (4)
N4—C3—H3A	109.1	Cl1—C7—Cl3	107.8 (3)
N3—C3—H3B	109.1	Cl2'—C7—Cl3	130.4 (5)
N4—C3—H3B	109.1	O2—C8—O1	127.3 (3)
H3A—C3—H3B	107.8	O2—C8—C7	119.2 (3)
N4—C4—N1	111.6 (3)	O1—C8—C7	113.5 (3)

Symmetry codes: (i) −*x*, *y*, −*z*+1/2.

### Hydrogen-bond geometry (Å, °)

**Table:**

*D*—H···*A*	*D*—H	H···*A*	*D*···*A*	*D*—H···*A*
C1—H1A···O2^ii^	0.97	2.52	3.416 (5)	153

Symmetry codes: (ii) −*x*, *y*+1, −*z*+1/2.
